# Impact of pulse pressure on clinical outcome in extracorporeal cardiopulmonary resuscitation (eCPR) patients

**DOI:** 10.1007/s00392-021-01838-7

**Published:** 2021-03-29

**Authors:** Jonathan Rilinger, Antonia M. Riefler, Xavier Bemtgen, Markus Jäckel, Viviane Zotzmann, Paul M. Biever, Daniel Duerschmied, Christoph Benk, Georg Trummer, Klaus Kaier, Christoph Bode, Dawid L. Staudacher, Tobias Wengenmayer

**Affiliations:** 1 Department of Medicine III (Interdisciplinary Medical Intensive Care), Medical Center, Faculty of Medicine, University of Freiburg, Freiburg, Germany; 2 Department of Cardiology and Angiology I, Faculty of Medicine, Heart Center Freiburg University, University of Freiburg, Hugstetterstr. 55, 79106 Freiburg, Germany; 3 Department of Cardiovascular Surgery, Faculty of Medicine, Heart Center Freiburg University, University of Freiburg, Freiburg, Germany; 4 Institute of Medical Biometry and Statistics, Faculty of Medicine, University Medical Center Freiburg, University of Freiburg, Freiburg, Germany

**Keywords:** Pulse pressure, Extracorporeal cardiopulmonary resuscitation, eCPR, Veno-arterial extracorporeal membrane oxygenation, Outcome

## Abstract

**Background:**

Hemodynamic response to successful extracorporeal cardiopulmonary resuscitation (eCPR) is not uniform. Pulse pressure (PP) as a correlate for myocardial damage or recovery from it, might be a valuable tool to estimate the outcome of these patients.

**Methods:**

We report retrospective data of a single-centre registry of eCPR patients, treated at the Interdisciplinary Medical Intensive Care Unit at the Medical Centre, University of Freiburg, Germany, between 01/2017 and 01/2020. The association between PP of the first 10 days after eCPR and hospital survival was investigated. Moreover, patients were divided into three groups according to their PP [low (0–9 mmHg), mid (10–29 mmHg) and high (≥ 30 mmHg)] at each time point.

**Results:**

One hundred forty-three patients (age 63 years, 74.1% male, 40% OHCA, average low flow time 49 min) were analysed. Overall hospital survival rate was 28%. A low PP both early after eCPR (after 1, 3, 6 and 12 h) and after day 1 to day 8 was associated with reduced hospital survival. At each time point (1 h to day 5) the classification of patients into a low, mid and high PP group was able to categorize the patients for a low (5–20%), moderate (20–40%) and high (50–70%) survival rate. A multivariable analysis showed that the mean PP of the first 24 h was an independent predictor for survival (*p* = 0.008).

**Conclusion:**

In this analysis, PP occurred to be a valuable parameter to estimate survival and maybe support clinical decision making in the further course of patients after eCPR.

**Graphic abstract:**

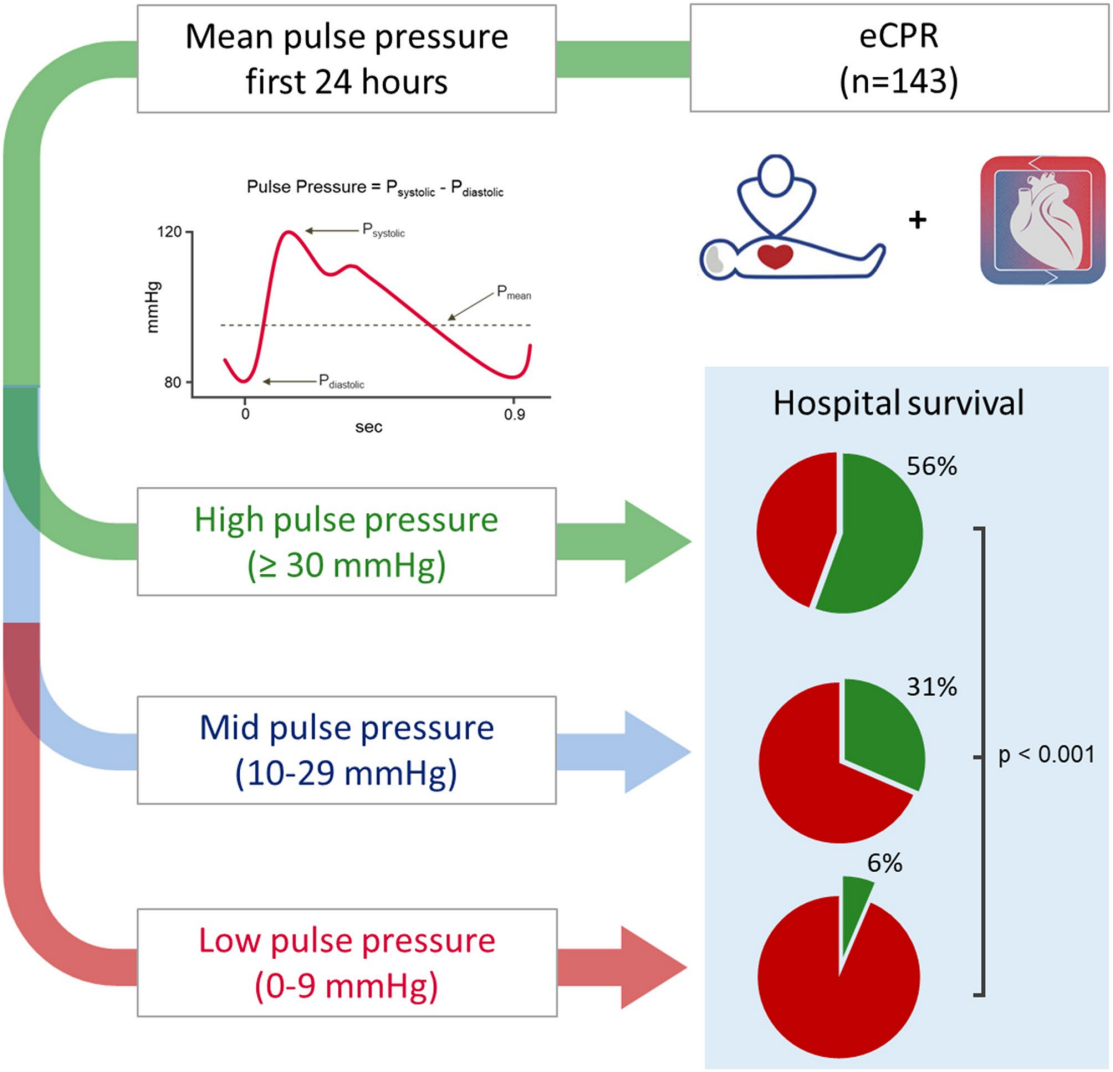

**Supplementary Information:**

The online version contains supplementary material available at 10.1007/s00392-021-01838-7.

## Background

More than 500,000 patients per year suffer cardiac arrest in the US [1] and approximately 275,000 out of hospital cardiac arrests (OHCA) per year are reported for Europe [2]. In case of a refractory cardiac arrest, veno-arterial extracorporeal membrane oxygenation (VA ECMO) support can be considered as a rescue therapy in these patients [3, 4], referred as extracorporeal cardiopulmonary resuscitation (eCPR) [5, 6]. Both, the increasing availability of VA ECMO systems and the encouraging results of previous studies lead to increasing eCPR numbers [7, 8].

Chances of survival are higher in eCPR patients (28–38%) [4, 9–12] than in patients treated with conventional cardiopulmonary resuscitation (CPR) (11–14%) [13, 14]. However, mortality remains high in these very sick patients. Although eCPR represents a promising approach for patients with cardiac arrest, this highly invasive, costly and complex therapy raises many new medical and ethical questions in which effective prognostic assessment plays an important role. So far, superior survival has been demonstrated for an initially defibrillatable heart rhythm, a shorter low flow time as well as a higher pH and a lower serum lactate level [15, 16].

Multiple scores have been established to estimate the possibility of survival after eCPR. Most of these scores rely on parameters from a time point before ECMO initiation [17] and do include the above mentioned parameters.

VA ECMO therapy aims on delivering adequate organ perfusion preventing worsening the post resuscitation syndrome and progression to multi-organ failure. Several biomarkers (e.g. lactate, pH and standard bicarbonate concentration) reflecting hemodynamic stabilization have been shown to prognosticate survival early on the treatment course [18].

Another evident marker of changes in hemodynamic state is the pulse pressure (PP), reflecting the myocardial function and the level of afterload [19]. In several non-ECMO studies the level of PP was investigated with regards to patient’s outcome, obtaining very different results. For instance, a high PP was associated with lower survival rates in patients with cardiogenic shock in case of an acute coronary syndrome and after myocardial infarction as well as after coronary artery bypasses graft surgery [20–22]. On the other hand, in patients with decompensated heart failure a low PP was a strong predictor for reduced survival [23].

Although it has been shown that PP is able to predict cardiac output in VA ECMO patients [24], so far there is little evidence on the prognostic value of PP in eCPR patients, especially while ongoing VA ECMO support. Therefore, this retrospective study investigated the influence of PP on the successful ECMO weaning and hospital survival rate within the first 10 days after eCPR.

## Methods

We report retrospective data of a single-centre registry of patients treated with eCPR. ECPR was defined as VA ECMO implantation during continuous resuscitation or within the first 20 min after return of spontaneous circulation (ROSC) with persistent hemodynamic instability [5]. Decision criteria that support or oppose the use of eCPR in the individual case and the further diagnostic and therapeutic strategy immediately after VA ECMO cannulation were performed in accordance with the German eCPR recommendations [6].

All patients treated at the Interdisciplinary Medical Intensive Care Unit at the Medical Centre, University of Freiburg, Germany, between January 2017 and January 2020 were analysed. Patient identity data derived from the registry were blinded and the study plan was approved by the local ethics committee (EK-Freiburg 151/14). The need for informed consent of the subjects was waived by the local ethics committee.

PP as well as all other analysed parameters were obtained from the medical patient records. Because of diagnostic transfers after VA ECMO implantation (CT scans or coronary angiography) immediately after eCPR, some PP values are missing (additional file 1, Table E7). Patients with missing values at a certain time point were not considered in the survival analysis. For visualization, the course of patients over time in relation to their corresponding pulse pressure PP was extrapolated using the next time-wise available value. After investigating the association between PP and hospital survival, patients were divided into three groups according to their median PP [low (0–9 mmHg), mid (10–29 mmHg) and high (≥ 30 mmHg)].

The primary endpoint was hospital survival. Moreover, successful VA ECMO weaning, 30 day survival and intensive care unit (ICU) survival were analysed. Successful ECMO weaning was defined as free from VA ECMO and alive for at least 48 h after decannulation. Unsuccessful weaning was defined as the inability to explant the ECMO device because of persistent cardiac failure or death during VA ECMO support or the need for re-cannulation within 48 h. To investigate pulse pressure independently from death due to anoxic brain injury, we also performed a survival analysis excluding these patients.

To compare the patient’s disease severity, the SAPS II score [25] at ICU admission was analysed.

Since, given the same level of diastolic blood pressure, there is collinearity for mean and systolic blood pressure as well as PP, only PP was considered in the multivariable analysis.

### Study population

In this study, only patients with eCPR (VA ECMO cannulation in case of cardiac arrest or instable ROSC) were investigated. Patients with VA ECMO cannulation in case of cardiogenic shock were excluded. All patients admitted to our ICU after eCPR were included in this study. At our institution, patients with in hospital cardiac arrest (IHCA) without ROSC after 15 min were routinely accessed for extracorporeal resuscitation. By local standard, the presence of an unwitnessed cardiac arrest, prolonged duration of CPR without signs of life (breathing, swallowing etc.), a non-shockable initial rhythm and advanced age were considered relative contraindications for VA ECMO cannulation. Final decision to cannulate, however, was driven by a team decision at the bedside including at least two physicians, a perfusionist and two nurses. For OHCA, emergency medical services personal was encouraged to transport patients without ROSC with ongoing chest compressions to the hospital. Since 09/2018 patients with OHCA without ROSC were evaluated for prehospital eCPR. The same relative contra indications applied as for IHCA patients. Cannulation was performed immediately on-site. All patients with evidence of a cardiac cause of the cardiac arrest were primarily assessed by coronary angiography after eCPR and then received a CT scan. Patients with suggestive symptoms or findings of an extra-cardiac cause received a CT scan first. In this case coronary angiography was performed if the CT scan had not provided a sufficient explanation for the cardiac arrest.

### ECMO centre and ECMO management

Our institution features a 24/7 ECMO-centre localized within a tertiary hospital with a 30-bed medical intensive care unit. Cannulations in our ECMO centre are performed by two experienced intensivist and a perfusionist in Seldinger’s technique without primary surgical cut down. All members of the ECMO team can be gathered within 30 min. Typical numbers for veno-arterial and veno-venous cannulation are 65 and 35 per year, respectively. There is a 24 h/7 days outreach team.

Either SCPC (Sorin centrifugal pump console, Livanova, London, United Kingdom) or Cardiohelp systems (Maquet Getinge Group, Rastatt, Germany) were used. Typical venous (draining) cannulas were 21–23 Fr (French = Charrière) in diameter and 55 cm of length while arterial (returning) cannulas were 15–17 Fr and 15–23 cm (both HLS cannula, Maquet Getinge Group, Rastatt, Germany). Since January 2019 decannulation is performed under fluoroscopic control using the MANTA Vascular Closure Device^®^ [26], whenever possible.

For patients without life threatening bleeding, anticoagulation was provided by intravenous unfractionated heparin aiming at a partial thromboplastin time 1.5 times upper normal limit.

The management of vasopressors and fluid therapy was driven by clinical judgement of the ECMO experienced intensivist in charge and has been reported earlier [27, 28]. Within the first 48 h of ECMO support blood flow was kept the highest possible flow without the need for excessive volume substation. All patients received invasive blood pressure monitoring via the right radial artery and cerebral NIRS monitoring.

Moreover, within the first 48 h of ECMO support no inotropes were applied if there was detectable pulse pressure. Only in case of severe pulmonary edema or if echocardiography showed no opening of the aortic valve inotropes therapy was started. In particularly severe cases, in which despite these measures, severe pulmonary edema or insufficient opening of the aortic valve persisted, mechanical left heart decompression using the Impella^®^ device was performed. The rationale of this algorithm is to avoid additional mechanical support to minimise additional complications. In one case intra-aortic balloon pump (IABP) was placed in addition to the VA ECMO therapy.

Treatment algorithms and standard operating procedures were subject to optimizations during the observational period, reflecting current state of the art recommendations and scientific knowledge.

### Statistical analysis

Continuous variables are presented as median and interquartile range (IQR) and categorical variables as numbers and percentages. Mann–Whitney *U* test was used for the analysis of continuous variables, Pearson’s Chi-squared test or Fisher’s exact test for categorical variables. Kruskal–Wallis test was used for the analysis of continuous variables in more than two groups. Multivariable regression analysis was performed for univariate (dependent) predictors of hospital survival (threshold of *p* < 0.05). Results are given as odds ratio [(OR), 95% confidence interval (CI)]. 60-day survival was calculated using the Kaplan–Meier method. Analyses were exploratory in nature. As a result, there was no prespecified plan to adjust for multiple comparisons and inferences drawn from 95% confidence intervals or *p* values may not be reproducible. Statistical calculations were performed using IBM SPSS statistics 25.0 (Armonk, NY: IBM Corp, 2017). Survival analysis was conducted in R 32 and figures were produced using the package ggplot2 33.

## Results

### Patients

**Fig. 1:**
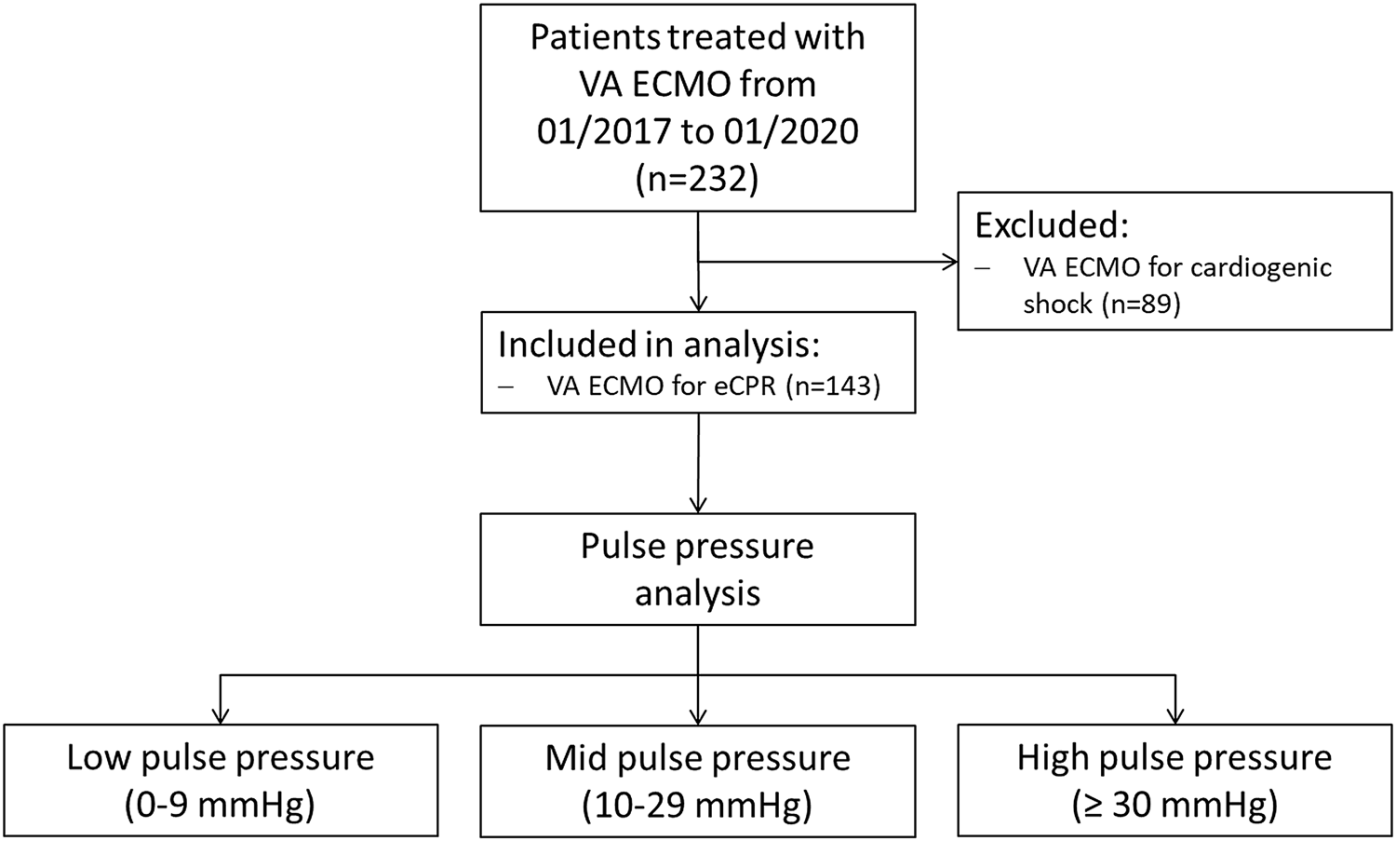
Study flow chart. Pulse pressure was analysed in 143 patients after eCPR. Patients were allocated to a low-, mid- and high-pulse pressure group for outcome prediction. *ECMO* extracorporeal membrane oxygenation, *eCPR* extracorporeal cardiopulmonary resuscitation, *PP* pulse pressure and *VA* veno-arterial

Two hundred thirty-two patients were treated with VA ECMO from 01/2017 to 01/2020. After exclusion of 89 patients treated for cardiogenic shock, 143 patients with eCPR (age 63 years, 74.1% male) could be analysed (Fig. 1). VA ECMO cannulation was performed in 135 patients while CPR and in 8 patients with unstable ROSC.

OHCA occurred in 39.9% of the patients and in 60.1% cardiac arrest had a coronary genesis (Table 1). Median no flow and low flow time were 0 min and 49 min, respectively. In 12 (8.4%) patients, eCPR was performed prehospital and 42% of all patients showed a shockable rhythm before VA ECMO implantation (additional file 1, table E1). SAPS II at ICU admission was 56.

**Table 1:** Baseline characteristics of the low-, mid- and high-pulse pressure groups

	All patients (*n* = 143)	Low PP (0–9 mmHg, *n* = 53)	Mid PP (10–29 mmHg, *n* = 54)	High PP (≥ 30 mmHg, *n* = 36)	*p* value
Age (years)	63 (54–73)	61 (49–70)	65 (58–73)	65 (57–76)	**0.040**
Sex (male)	106 (74.1%)	40 (75.5%)	37 (68.5%)	29 (80.6%)	0.425
BMI (kg/m^2^)	25 (24–28)	24 (24–28)	25 (24–28)	25 (24–28)	0.575
Location of cardiac arrest					**0.013**
IHCA	86 (60.1%)	24 (45.3%)	35 (64.8%)	27 (75%)	
OHCA	57 (39.9%)	29 (54.7%)	19 (35.2%)	9 (25%)	
Cause of cardiac arrest					0.341
Coronary	86 (60.1%)	35 (66%)	33 (61.1%)	18 (50%)	
Cardiac, non-coronary	32 (22.4%)	10 (18.9%)	14 (25.9%)	8 (22.2%)	
Other	25 (17.5%)	8 (15.1%)	7 (13%)	10 (27.8%)	
No flow time pre VA ECMO (min)	0 (0–0)	0 (0–2.8)	0 (0–0)	0 (0–0)	0.166
Low flow time pre VA ECMO (min)	49 (30–70)	55 (35–75)	44 (25–70)	43 (29–64)	0.115
SAPS II at ICU admission	56 (48–64)	61 (54–68)	53 (46–62)	52 (46–61)	**0.004**
Lactate 3 h after VA ECMO (mmol/l)	7.5 (4.1–11.6)	7 (4.1–11.6)	7.7 (2.6–11)	10.3 (4.8–17)	0.635
Creatine kinase (U/l)	589 (214–2832)	1107 (244–6762)	561 (166–1783)	361 (177–851)	**0.015**
Creatine kinase—MB (U/l)	182 (105–525)	273 (122–819)	163 (120–400)	105 (56–258)	**0.018**
Myoglobin (ng/ml)	1385 (399–4795)	3026 (413–9015)	1373 (407–3520)	995 (337–1936)	0.138
Comorbidities					
Coronary artery disease	52 (36.4%)	14 (26.4%)	19 (35.2%)	19 (52.8%)	**0.039**
Chronic heart failure	22 (15.4%)	6 (11.3%)	7 (13%)	9 (25%)	0.176
Chronic renal failure	25 (17.5%)	6 (11.3%)	8 (14.8%)	11 (30.6%)	0.052
Liver cirrhosis	12 (8.4%)	1 (1.9%)	7 (13%)	4 (11.1%)	0.094
Pulmonary diseases	27 (18.9%)	10 (18.9%)	8 (14.8%)	9 (25%)	0.481

*p* values < 0.05 are presented in bold.

Table shows baseline characteristics of the pulse pressure groups (low, mid and high) for patients alive after 24 h. Mean pulse pressure of the first 24 h was used for group definition

*BMI* body mass index, *ECMO* extracorporeal membrane oxygenation, *ICU* intensive care unit, *IHCA* in hospital cardiac arrest, *OHCA* out of hospital cardiac arrest, *PP* pulse pressure, *SAPS II score* Simplified Acute Physiology Score and *VA* veno-arterial

Overall successful VA ECMO weaning and hospital survival rate were 35.7% and 28%, respectively (Table 2). Six (4.2%) patients received additional Impella^®^ support and one (0.7%) patient additional IABP support.

**Table 2:** Procedural characteristics and outcome of the low-, mid- and high-pulse pressure groups

	All patients (*n* = 143)	Low PP (0–9 mmHg, *n* = 53)	Mid PP (10–29 mmHg, *n* = 54)	High PP (≥ 30 mmHg, *n* = 36)	*p* value
Successful ECMO weaning	51 (35.7%)	8 (15.1%)	21 (38.9%)	22 (61.1%)	** < 0.001**
30 day survival	44 (30.8%)	5 (9.4%)	18 (33.3%)	21 (58.3%)	** < 0.001**
ICU survival	42 (29.4%)	4 (7.5%)	18 (33.3%)	20 (55.6%)	** < 0.001**
Hospital survival	40 (28%)	3 (5.7%)	17 (31.5%)	20 (55.6%)	** < 0.001**
Additional Impella^®^ support	6 (4.2%)	4 (7.6%)	2 (3.7%)	0 (0%)	0.134
Additional IABP support	1 (0.7%)	0 (0%)	1 (1.8%)	0 (0%)	0.134
Norepinephrine mean 24 h (µg/kg BW/min)	0.4 (0.2–0.5)	0.4 (0.2–0.7)	0.2 (0.1–0.5)	0.3 (0.2–0.5)	0.575
Epinephrine mean 24 h (µg/kg BW/min)	0 (0–0.1)	0 (0–0.4)	0 (0–0.1)	0 (0–0.1)	0.642
Dobutamine mean 24 h (µg/kg BW/min)	0 (0–0)	0 (0–0)	0 (0–0)	0 (0–29.2)	0.436
ICU length of stay (days)	4.8 (1.3–12.5)	1.9 (0.4–7.6)	4.8 (2–11.9)	8.1 (5.3–20.4)	** < 0.001**
VA ECMO duration (days)	2.8 (0.8–4.8)	1.3 (0.4–4.3)	3.1 (1.6–5.1)	2.9 (0.9–4.9)	0.032

*p* values < 0.05 are presented in bold.

Mean pulse pressure of the first 24 h was used for group definition

*BW* body weight, *ECMO* extracorporeal membrane oxygenation, *IABP* intra-aortic balloon pump, *ICU* intensive care unit, *PP* pulse pressure and *VA* veno-arterial

### Pulse pressure analysis

Throughout from 1 h to day 8 after eCPR, a low pulse pressure correlated with reduced hospital survival (additional file 1, table E2). A similar association was observed between low PP and reduced VA ECMO weaning rate (additional file 1, table E3). There was no difference in measured PP pre VA ECMO implantation (while CPR) in survivors and non-survivors (only applicable for patients with unstable ROSC).

The classification of patients into a low (0–9 mmHg), mid (10–29 mmHg) and high (≥ 30 mmHg) PP group showed a good discrimination between low, medium and high survival rates at each time point from 1 h until day 5 after eCPR (Table 3). The same association was shown in the subgroup of patients with ongoing VA ECMO only at each time point (additional file 1, table E4).

**Table 3:** Survival rates of patients with low-, mid- and high-pulse pressure at different time points

	Low PP (0–9 mmHg)	Mid PP (10–29 mmHg)	High PP (≥ 30 mmHg)	*p* value
PP pre implant	31 (28.4%)	0 (0%)	4 (33.3%)	0.158
PP 1 h	1 (8.3%)	5 (21.7%)	11 (50%)	**0.022**
PP 3 h	9 (22.5%)	12 (24.5%)	15 (50%)	**0.024**
PP 6 h	8 (14%)	16 (39%)	16 (55.2%)	** < 0.001**
PP 12 h	9 (17.6%)	16 (38.1%)	14 (56%)	**0.003**
PP 24 h	4 (10.3%)	19 (52.8%)	17 (51.5%)	** < 0.001**
PP mean of the first 24 h	3 (6.4%)	17 (31.5%)	20 (55.6%)	** < 0.001**
PP d2	1 (6.7%)	8 (26.7%)	31 (66%)	** < 0.001**
PP d3	0 (0%)	5 (26.3%)	35 (62.5%)	** < 0.001**
PP d5	0 (0%)	4 (28.6%)	36 (66.7%)	**0.009**
PP d7	0 (0%)	2 (40%)	35 (71.4%)	0.127

*p* values < 0.05 are presented in bold.

Hospital survival rates are shown pre implant and for advancing time periods after eCPR for the three pulse pressure groups

*eCPR* extracorporeal cardiopulmonary resuscitation and *PP* pulse pressure

**Fig. 2:**
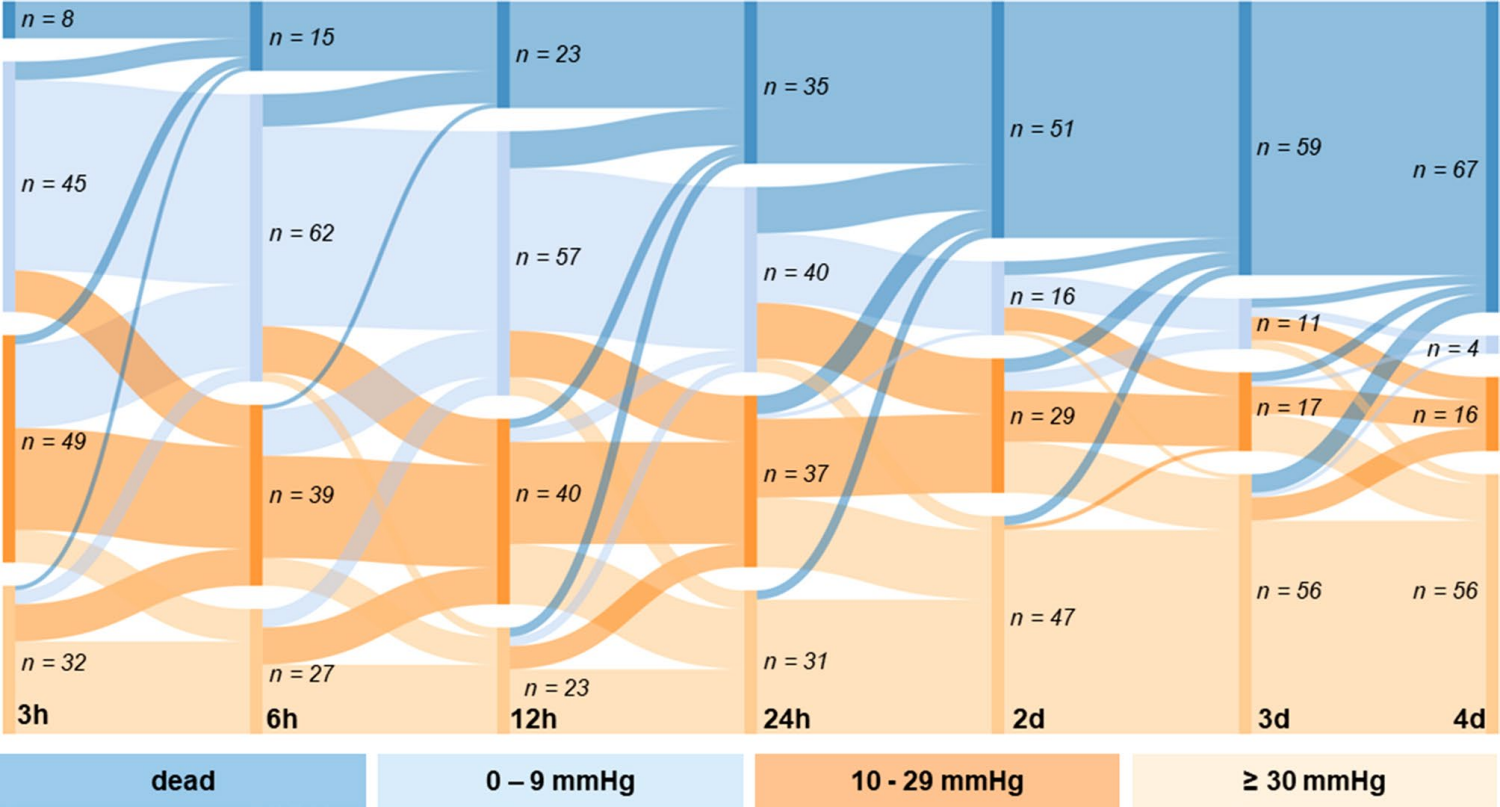
Sankey chart displaying the course of patients over the time in relation to their corresponding pulse pressure. *eCPR* extracorporeal cardiopulmonary resuscitation and *PP* pulse pressure

Although low or high PP at each single time point showed a strong association to increased mortality or survival rates, many patients switched between the different PP allocations in the first 24 h (Fig. 2). The Sankey chart visualizes the course of patients over the time in relation to their corresponding pulse pressure. Particularly, a large proportion of patients had alternating low or mid PP in the first 24 h. At each time point a high number of patients presenting with a low PP were dying, while mortality of patients with a high PP was very low. All over, the proportion of deceased patients and patients with a high PP was constantly increasing while the proportion of patients with a low or mid PP was decreasing.

### Analysis of mean 24 h pulse pressure

There were no differences between the low, mid and high PP groups in sex and BMI, but patients with a low PP were younger (61 vs. 65 and 65 years, *p* = 0.040, Table 1). Moreover, patients with a low PP showed a higher rate of OHCA (55% vs. 35% and 25%, *p* = 0.013) compared to patients with a mid and high PP. The underlying cause of cardiac arrest was similar in all three groups, with 50–60% of patients showing a coronary cause. Low PP patients showed higher creatine kinase and creatine kinase-MB levels than patients with mid and high PP (*p* = 0.015 and *p* = 0.018).

There were no differences in the no flow time between the three groups, but patients with a low PP showed a numerically but not statistically significant higher low flow time (55 min vs. 44 min and 43 min). Moreover, SAPS II at hospital admission was higher in the low PP group. Interestingly, the rate of underlying coronary artery disease was higher in the mid and high PP group (*p* = 0.039). Furthermore, there was a trend for a higher rate of chronic renal failure in patients with a high PP (*p* = 0.052).

**Fig. 3:**
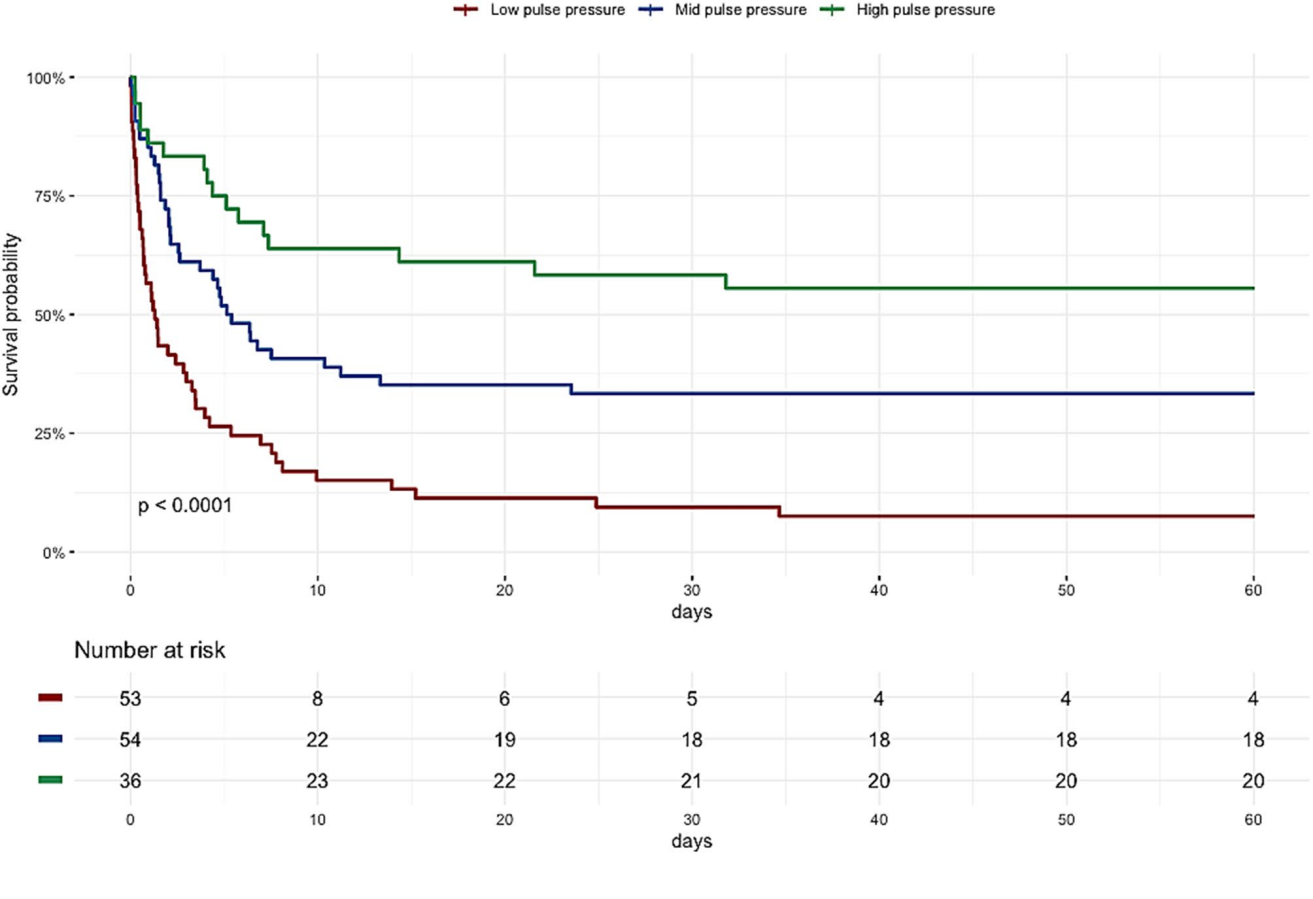
Hospital survival of eCPR patients divided by mean 24 h pulse pressure. Log Rank test: *p* < 0.001. *eCPR* extracorporeal cardiopulmonary resuscitation and *PP* pulse pressure

Successful VA ECMO weaning rate was higher for patients with a high PP (61%) than for patients with mid (39%) and low PP (15%, *p* < 0.001, Table 2). Hospital survival rates for patients with a mean low, mid and high PP in the first 24 h after eCPR were 5.7%, 31.5% and 55.6%, respectively (*p* < 0.001, Fig. 3). After exclusion of patients died due to anoxic brain injury or withdrawal of care in those circumstances, survival rates were 7.9%, 42.5% and 71.4%, respectively (*p* < 0.001). Survival rates of PP groups in patients without additional mechanical circulatory support (Impella^®^ or IABP) were 6.4%, 31.4% and 55.6%, respectively (*p* < 0.001).

### Analysis of pulse pressure as an independent predictor for survival

Univariate analysis showed a strong association of high mean PP of the first 24 h, high mean and systolic arterial pressure as well as a higher level of pH and standard bicarbonate, a lower level of potassium, glucose and lactate 24 h after eCPR with increased survival (additional file 1, table E5). Interestingly, diastolic arterial pressure was not associated with survival.

Multivariable analysis revealed mean PP of the first 24 h after eCPR [OR 3.2 (1.3–7.4), *p* = 0.008] and lactate [OR 0.6 (0.4–0.8), *p* = 0.003] as independent predictor for survival (Table 4).

**Table 4:** Multivariate prognostic analysis 24 h after eCPR

	OR (95%CI)	*p* value
PP mean of the first 24 h (mmHg)	3.2 (1.3–7.4)	**0.008**
pH	3.0 (0 to > 100)	0.795
Lactate (mmol/l)	0.6 (0.4–0.8)	**0.003**
Standard bicarbonate (mmol/l)	0.9 (0.7–1.3)	0.623
Potassium (mmol/l)	0.4 (0.2–1.3)	0.141
Glucose (mg/dl)	1.0 (1.0–1.0)	0.236

*p* values < 0.05 are presented in bold.

Table shows results of a multivariate analysis of the mean PP of the first 24 h and parameters of blood gas analysis 24 h after eCPR which were associated with increased or reduced hospital survival in a univariate prognostic analysis

*eCPR* extracorporeal cardiopulmonary resuscitation and *PP* pulse pressure

## Discussion

To our best knowledge, this is the first comprehensive analysis of PP and its meaning for the clinical course of eCPR patients. In this analysis low PP after eCPR was strongly associated with a reduced survival rate. The level of mean PP of the first 24 h corresponded to a survival rate of 6%, 32% and 56%, respectively. The discrimination between these three groups was present from the first hours after eCPR and lasted up to day 5.

The patients in this study showed similar baseline characteristics compared to previous reports from eCPR cohorts. This is especially true for important prognostic factors like low flow time, shockable heart rhythm and coronary genesis of cardiac arrest [10, 11, 15]. In addition, this analysis included patients with IHCA, OHCA and pre hospital VA ECMO cannulation, which covers the entire spectrum of eCPR applications. Furthermore, this cohort showed a rather high survival and VA ECMO weaning rate.

The allocation into a low (0–9 mmHg), mid (10–29 mmHg) and high (≥ 30 mmHg) PP group for each time point was based on clinical considerations and resulted in three risk groups. Patients with a low PP showed a survival rate of approx. 5–20% for each time point and patients with mid and high PP showed a survival rate of approx. 20–40% and 50–70%, respectively. Thus, not only an association between a very low PP and a high mortality could be shown, but also an association between a high PP and an above the average high survival rate compared to the overall cohort and other eCPR reports (30–40% survival rate) [4, 9–11]. In a subgroup analysis excluding patients who died of anoxic brain injury, these group differences were even more pronounced, suggesting a possible influence of PP on the physiology and organ function in these patients.

To extend the isolated observation of PP at each single time point, an analysis of the course of PP of all patients within the first days after eCPR was performed. The Sankey chart analysis reveals that patients do not remain permanently in a PP group, but change between the groups to a relevant extent, especially within the first 24 h. As the survival analysis of the three PP groups at each time point suggests, the rate of patients dying is highest in the low PP group. Furthermore, most of the changes between the groups were completed within the first 4 days, so that afterwards most of the patients already died or showed a recovery of the PP and thus changed into the high PP group.

Both from a clinical point of view and due to the high proportion of patients switching between PP groups within the first 24 h, a detailed analysis of the mean PP of the first 24 h was performed to examine the three PP groups with regard to their baseline characteristics and outcome.

There were several factors that were associated with a low PP level. First of all, patients with a low PP had a higher rate of OHCA compared to patients with mid and high PP. Corresponding to a higher rate of OHCA, the low PP group also showed an at least numerically higher low flow time (10 min longer than patients with mid and high PP). Moreover, patients with a low PP showed higher levels of creatine kinase and creatine kinase—MB. Therefore, the distribution of the duration of low flow time and creatine kinase might be appropriate to explain the level of PP as an indirect parameter for myocardial damage. On the other hand, PP is also determined by the level of arterial afterload. Therefore, it is important to note, that there were neither differences in the applied dose of the inotropic nor the vasoconstrictive therapy between the three PP groups.

In a few patients (6, 4.2%) with no opening of the aortic valve after inotropic stimulation or in case of severe pulmonary edema, the left heart decompression device Impella^®^ or IABP were used additionally to the VA ECMO therapy. Even though there was no significant difference of left ventricular venting between the groups, this additional support may have led to a further reduced PP. These patients certainly represent a special cohort with an increased risk of mortality, but it seems reasonable to include these patients in this study as well, as it corresponds to real life experience and therapy. Moreover, even after excluding these patients from the analysis, the same association between PP and survival was observed.

Interestingly, there were no differences in no flow time, cause of cardiac arrest or underlying heart rhythm between the three groups. And paradoxically, there was a significantly higher rate of underlying coronary artery disease and a trend for a higher rate of chronic renal failure in patients from the high PP group, which might be explained by the higher rate of IHCA, as the patients were already hospitalised with corresponding diagnoses.

Beyond the extent of a strong association between the PP level and patient outcomes, the mean 24 h PP appeared as an independent predictor for survival besides the lactate level in a multivariable analysis of parameters available at bedside (vital parameters and blood gas analysis).

The results of this work thus represent a continuation of the findings of previous studies that examined the PP at the time before, during or immediately after VA ECMO cannulation.

Previously, it could be shown that PP before VA ECMO cannulation is an important prognostic parameter in patients with a cardiogenic shock. The survival after veno-arterial ECMO (SAVE) score includes a PP of ≤ 20 mmHg as a factor for increased in mortality [17]. Appropriately, the ECPR score by Park et al*.* included an initial PP immediately after VA ECMO cannulation of ≥ 24 mmHg as a factor for a higher survival rate [29]. These results are confirmed by a study of Ryu et al*.* in which an initial pulse pressure after eCPR of less than 25 mmHg was associated with inferior neurological outcome [30].

The only study that investigated the influence of the PP in the further course after eCPR, examined the period of the first 6 h after VA ECMO cannulation. In this mixed cohort (cardiogenic shock, respiratory failure and septic shock) of 69 patients, a high mean PP (≥ 30 mmHg) was associated with a superior survival and VA ECMO weaning rate [31].

The presented results show for the first time that the prognostic value of the PP is not only present at the time immediately before and after eCPR, but also at any other time within the next 5 days. In summary, we believe that the PP after eCPR, a parameter that is easy to assess at any time, has a high predictive value for survival. Although the PP should not be considered on its own and the shown results should be confirmed in larger trials, it certainly may help in clinical decisions making.

## Limitations

This is a retrospective observational study and, therefore, contains the risk of selection and reporting bias.

Obviously, PP is not only driven by the patient’s capability to produce a PP but depends on medical or device treatment. In this analysis inotropic support was similar between the groups and only very few venting devices were used. For that, these confounders should be addressed adequately. However, the present observations can be transferred only to eCPR cohorts with similar treatment regimes.

Therefore, despite of using multivariable analysis to prove PP as an independent predictor for survival, there still might be remaining confounders. Moreover, this is a single-centre report and specific processes may influence the presented results. Together, due to these limitations, our findings about the PP after eCPR should be considered in the overall context of the patient's disease severity and should not prompt therapy limitations on its own.

Furthermore, for the results of the first 12 h it must be taken into account that due to patient transports and interventions some PP values were missing.

## Conclusion

In this analysis, PP after eCPR was found to be a strong and independent predictor for survival, which could potentially be a useful parameter in daily clinical decision making.

## Supplementary Information

Below is the link to the electronic supplementary material.

Supplementary file1 (DOCX 167 KB)

## Data Availability

The datasets used and/or analysed during the current study are available from the corresponding author on reasonable request.

## Abbreviations

APACHE II score: Acute physiology and chronic health evaluation
BMI: Body mass index
BW: Body weight
CPR: Cardiopulmonary resuscitation
ECMO: Extracorporeal membrane oxygenation
eCPR: Extracorporeal cardiopulmonary resuscitation
IABP: Intra-aortic balloon pump
IHCA: In hospital cardiac arrest
ICU: Intensive care unit
OHCA: Out of hospital cardiac arrest
PP: Pulse pressure
PEA: Pulseless electrical activity
ROSC: Return of spontaneous circulation
SAPS II score: Simplified Acute Physiology Score
UHF: University Hospital Freiburg
VA: Veno-arterial
