# Supplementary material for: Impact of pulse pressure on clinical outcome in extracorporeal cardiopulmonary resuscitation (eCPR) patients
Source: Clin Res Cardiol. 2021 Mar 29;110(9):1473–83. doi: 10.1007/s00392-021-01838-7 (PMC8405467; doi:10.1007/s00392-021-01838-7)
Supplement: Supplementary file 1 — Supplementary file1 (DOCX 167 KB) [file 392_2021_1838_MOESM1_ESM.docx]

**Pulse pressure after extracorporeal cardiopulmonary resuscitation (eCPR) and its impact on survival**

Jonathan Rilinger, Antonia M Riefler, Xavier Bemtgen, Markus Jäckel, Viviane Zotzmann, Paul M Biever, Daniel Duerschmied, Christoph Benk, Georg Trummer, Klaus Kaier, Christoph Bode, Dawid L Staudacher, Tobias Wengenmayer

Categorical variables are presented as frequency (percentages). Continuous variables are presented as median (IQR).

## Tables

### Table E1. Additional information of the comparison of baseline characteristics for the low, mid and high pulse pressure groups (mean of the first 24 hours)

|  | **All patients**  **(n=143)** | **Low PP**  **(0-9mmHg, n=53)** | **Mid PP**  **(10-29mmHg, n=54)** | **High PP**  **(≥30mmHg, n=36)** | **P value** |
| --- | --- | --- | --- | --- | --- |
| Place of eCPR |  |  |  |  | 0.484 |
| University hospital  Freiburg | 125 (87.4%) | 45 (84.9%) | 48 (88.9%) | 32 (88.9%) |  |
| External hospital | 6 (4.2%) | 1 (1.9%) | 3 (5.6%) | 2 (5.6%) |  |
| Pre hospital | 12 (8.4%) | 7 (13.2%) | 3 (5.6%) | 2 (5.6%) |  |
| EKG rhythm pre eCPR |  |  |  |  | 0.764 |
| Asystole | 20 (14%) | 10 (18.9%) | 6 (11.1%) | 4 (11.1%) |  |
| PEA | 55 (38.5%) | 18 (34%) | 22 (40.7%) | 15 (41.7%) |  |
| Shockable rhythm | 60 (42%) | 22 (41.5%) | 22 (40.7%) | 16 (44.4%) |  |
| Unknown | 8 (5.6%) | 3 (5.7%) | 4 (7.5%) | 1 (2.8%) |  |

Table shows baseline characteristics of the pulse pressure groups (low, mid and high) for patients alive after 24 hours. Mean pulse pressure of the first 24 hours was used for group definition. *eCPR: extracorporeal cardiopulmonary resuscitation; PEA: Pulseless electrical activity; PP: pulse pressure.*

### Table E2. Association between pulse pressure and hospital survival

|  | **Patients alive** | **All patients** | **Survivors** | **Non-survivors** | **P value** |
| --- | --- | --- | --- | --- | --- |
| PP *pre implant* | 143 | 0 (0-0) | 0 (0-0) | 0 (0-0) | 0.417 |
| PP 1h | 143 | 23 (12-41) | 37 (26.5-51.5) | 20.5 (6.8-30.8) | **0.002** |
| PP 3h | 135 | 14 (7-30) | 19 (9.3-42.8) | 13 (6-24) | **0.021** |
| PP 6h | 128 | 12 (5-27) | 20 (12-41.5) | 8 (3-19) | **<0.001** |
| PP 12h | 120 | 12 (2.8-27) | 22 (12-38) | 8 (0-19) | **<0.001** |
| PP 24h | 108 | 16 (6-33) | 27 (14.5-41) | 8 (2.3-26.8) | **<0.001** |
| PP mean of the first 24h | 143 | 15.8 (7-31.6) | 30.6 (16.1-40.5) | 12 (4.8-20.5) | **<0.001** |
| PP d2 | 92 | 31 (15-54) | 50.5 (31.3-62.3) | 22.5 (8.3-34.8) | **<0.001** |
| PP d3 | 84 | 43 (23.3-60.8) | 58 (43-72) | 27 (10.3-43.8) | **<0.001** |
| PP d4 | 76 | 46 (30-60) | 56.5 (45-72.3) | 33 (23-49) | **<0.001** |
| PPd5 | 70 | 47.5 (33.5-69.3) | 59.5 (45-72.8) | 36 (23-45.3) | **<0.001** |
| PP d6 | 64 | 59 (41.3-72) | 63.5 (46.5-75.8) | 53 (25-63.5) | **0.016** |
| PP d7 | 55 | 58 (47-69) | 60 (52-75.5) | 44.5 (34.3-61.8) | **0.012** |
| PP d8 | 50 | 61 (52-77.3) | 69 (54.5-90) | 52 (40.5-63.5) | **0.013** |
| PP d9 | 47 | 63 (50-84) | 63 (51-85) | 61 (39.8-83.5) | 0.464 |
| PP d10 | 45 | 57 (49.5-75.5) | 57.5 (50.8-77) | 57 (45-72) | 0.457 |

Pulse pressure is presented in mmHg. *PP: pulse pressure.*

### Table E3. Association between pulse pressure and successful VA ECMO weaning

|  | **Patients alive** | **All patients** | **Successful weaning** | **Unsuccessful weaning** | **P value** |
| --- | --- | --- | --- | --- | --- |
| PP *pre implant* | 143 | 0 (0-0) | 0 (0-0) | 0 (0-0) | 0.479 |
| PP 1h | 143 | 23 (12-41) | 33.5 (20.5-49) | 20 (6-31) | **0.006** |
| PP 3h | 135 | 14 (7-30) | 20 (9-40) | 13 (6-23) | **0.023** |
| PP 6h | 128 | 12 (5-27) | 19 (9-37) | 7.5 (3-18.8) | **<0.001** |
| PP 12h | 120 | 12 (2.8-27) | 16.5 (6.8-33) | 8 (2-19.8) | **0.006** |
| PP 24h | 108 | 16 (6-33) | 27 (14-42) | 7 (2.5-25.5) | **<0.001** |
| PP mean of the first 24h | 143 | 15.8 (7-31.6) | 26.6 (13.8-38) | 11.9 (4.4-19.6) | **<0.001** |
| PP d2 | 92 | 31 (15-54) | 47 (27-60) | 17 (8-34) | **<0.001** |
| PP d3 | 84 | 43 (23.3-60.8) | 54 (34-71) | 24 (9.5-40.5) | **<0.001** |
| PP d4 | 76 | 46 (30-60) | 55 (42-64) | 29 (20.5-47) | **<0.001** |
| PPd5 | 70 | 47.5 (33.5-69.3) | 59 (44-72) | 28 (23-39) | **<0.001** |
| PP d6 | 64 | 59 (41.3-72) | 60 (46-75) | 45 (16.5-59.5) | **0.011** |
| PP d7 | 55 | 58 (47-69) | 60 (52-75) | 32.5 (18.3-37.8) | **<0.001** |
| PP d8 | 50 | 61 (52-77.3) | 65 (54-79.3) | 40.5 (22-50) | **0.004** |
| PP d9* | 47 | 66.1±24.2 | 68.8±22.3 | 26.7±18.7 | **0.002** |
| PP d10* | 45 | 60.4±19.9 | 61.4±19.8 | 39±8.5 | 0.073 |

* Mean ± standard deviation is shown because of low case numbers. Pulse pressure is presented in mmHg. *ECMO: extracorporeal membrane oxygenation; PP: pulse pressure; VA: veno-arterial.*

### Table E4. Hospital survival and VA ECMO weaning rates for patients with low-, mid and high pulse pressure and ongoing VA ECMO support

|  | **Patients on VA ECMO** | **Low PP**  **(0-9mmHg)** | **Mid PP**  **(10-29mmHg)** | **High PP**  **(≥30mmHg)** | **P value** |
| --- | --- | --- | --- | --- | --- |
| **Hospital survival** |  |  |  |  |  |
| 6h | 125 | 15 (20.5%) | 18 (43.9%) | 5 (45.5%) | **0.018** |
| 24h | 102 | 4 (10.3%) | 19 (52.8%) | 13 (48.1%) | **<0.001** |
| d3 | 65 | 0 (0%) | 5 (26.3%) | 20 (52.6%) | **0.009** |
| d5 | 32 | 0 (0%) | 2 (18.2%) | 8 (42.1%) | 0.243 |
| d7 | 14 | 0 (0%) | 0 (0%) | 3 (30%) | 0.466 |
| **Successful weaning** |  |  |  |  |  |
| 6h | 125 | 14 (28.6%) | 19 (38.8%) | 16 (32.7%) | **0.005** |
| 24h | 102 | 8 (17.4%) | 21 (45.7%) | 17 (37%) | **<0.001** |
| d3 | 65 | 1 (12.5%) | 8 (42.1%) | 25 (65.8%) | **0.013** |
| d5 | 32 | 1 (50%) | 3 (27.3%) | 13 (68.4%) | 0.093 |
| d7 | 14 | 0 (0%) | 0 (0%) | 6 (60%) | 0.122 |

*ECMO: extracorporeal membrane oxygenation; PP: pulse pressure; VA: veno-arterial.*

### Table E5. Univariate prognostic analysis of vital parameters and blood gas analysis for patients alive 24 hours after eCPR

|  | **Dead (n=68)** | **Survivors (n=40)** | **P value** |
| --- | --- | --- | --- |
| PP mean of the first 24h | 12 (4.8-20.5) | 30.6 (16.1-40.5) | **< 0.001** |
| Heart rate /min | 71 (58-89) | 66.5 (58-79) | 0.211 |
| Breathing rate /min | 15 (11-19) | 15 (14-17.3) | 0.939 |
| SpO2 (%) | 98 (94-100) | 98 (96.8-100) | 0.078 |
| Mean arterial pressure (mmHg) | 68 (64-80.8) | 79.5 (70.5-82) | **0.008** |
| Systolic arterial pressure (mmHg) | 79 (69-91.8) | 95 (83-115) | **0.000** |
| Diastolic arterial pressure (mmHg) | 66 (56.5-77.3) | 68.5 (61-73) | 0.368 |
| pO2 (mmHg) | 119 (85.9-243) | 110 (82.1-163.3) | 0.517 |
| pCO2 (mmHg) | 36.4 (33.7-41.3) | 37.7 (36-41.3) | 0.201 |
| pH | 7.35 (7.32-7.42) | 7.42 (7.35-7.45) | **0.032** |
| Standard bicarbonate (mmol/l) | 21 (18.3-24) | 23.5 (21.9-25.6) | **0.001** |
| Hb (g/dl) | 8.6 (7.8-10) | 8.8 (8.2-10.4) | 0.137 |
| Potassium (mmol/l) | 4.6 (4.3-5.1) | 4.4 (4.1-4.7) | **0.045** |
| Natrium (mmol/l) | 143 (139-148) | 143 (140.5-146) | 0.993 |
| Calcium (mmol/l) | 1.1 (1.1-1.2) | 1.1 (1.1-1.2) | 0.080 |
| Chloride (mmol/l) | 110 (107-114) | 112 (108.8-115) | 0.111 |
| Glucose (mg/dl) | 152 (116-173.5) | 132.5 (120.5-146.3) | **0.044** |
| Lactate (mmol/l) | 5.4 (2.7-9.3) | 2.4 (1.4-3.3) | **0.000** |
| Bilirubin (mg/dl) | 1.7 (1.2-2.8) | 1.6 (0.9-2.1) | 0.076 |
| Haematocrit (%) | 26.6 (24.3-30.7) | 27.4 (25.5-32.1) | 0.130 |

*eCPR: extracorporeal cardiopulmonary resuscitation; PP: pulse pressure.*

### Table E6. VA ECMO weaning rates of patients with low, mid and high pulse pressure

|  | **Low PP**  **(0-9mmHg)** | **Mid PP**  **(10-29mmHg)** | **High PP**  **(≥30mmHg)** | **P value** |
| --- | --- | --- | --- | --- |
| PP *pre implant* | 40 (36.7%) | 1 (11.1%) | 4 (33.3%) | 0.299 |
| PP 1h | 2 (16.7%) | 8 (34.8%) | 12 (54.5%) | 0.085 |
| PP 3h | 12 (30%) | 18 (36.7%) | 17 (56.7%) | 0.068 |
| PP 6h | 14 (24.6%) | 19 (46.3%) | 18 (62.1%) | **0.002** |
| PP 12h | 15 (29.4%) | 20 (47.6%) | 15 (60%) | **0.028** |
| PP 24h | 8 (20.5%) | 21 (58.3%) | 22 (66.7%) | **<0.001** |
| PP mean of the first 24h | 8 (17%) | 21 (38.9%) | 22 (61.1%) | **<0.001** |
| PP d2 | 2 (13.3%) | 13 (43.3%) | 36 (76.6%) | **<0.001** |
| PP d3 | 1 (11.1%) | 8 (42.1%) | 42 (75%) | **<0.001** |
| PPd5 | 1 (50%) | 5 (35.7%) | 45 (83.3%) | **0.001** |
| PP d7 | 0 (0%) | 2 (40%) | 45 (91.8%) | **<0.001** |

VA ECMO weaning rates are shown pre implant and for advancing time periods after eCPR for the three pulse pressure groups. *ECMO: extracorporeal membrane oxygenation; eCPR: extracorporeal cardiopulmonary resuscitation; PP: pulse pressure; VA: veno-arterial.*

### Table E7. Missing pulse pressure values

|  | **Missing values** |
| --- | --- |
| PP *pre implant* | 13 (9.1%) |
| PP 1h | 68 (47.6%) |
| PP 3h | 16 (11.9%) |
| PP 6h | 1 (0.8%) |
| PP 12h | 2 (1.7%) |

In the first 12 hours some pulse pressure values are missing because of diagnostic transfers after VA ECMO implantation (CT scans or coronary angiography). After the 12 hour period the documentation is gapless. *ECMO: extracorporeal membrane oxygenation; PP: pulse pressure; VA: veno-arterial.*

### Table E8. Hospital survival of the low-, mid and high pulse pressure groups* - exclusion of patients with withdrawal of care in case of severe anoxic brain injury

|  | **All patients**  **(n=106)** | **Low PP**  **(0-9mmHg, n=38)** | **Mid PP**  **(10-29mmHg, n=40)** | **High PP**  **(≥30mmHg, n=28)** | **P value** |
| --- | --- | --- | --- | --- | --- |
| Hospital survival | 40 (37.7%) | 3 (7.9%) | 17 (42.5%) | 20 (71.4%) | **<0.001** |

*Mean pulse pressure of the first 24 hours was used for group definition.

## Figures


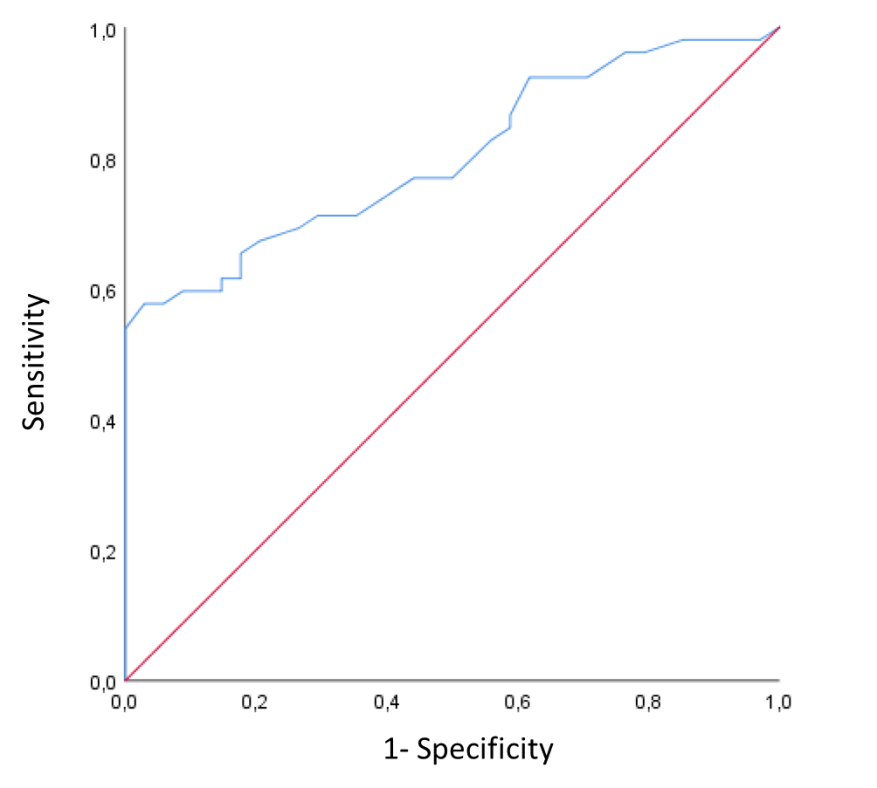


### Figure E1. AUC of independent predictor – lactate 24 hours after eCPR

AUC = 0.80.


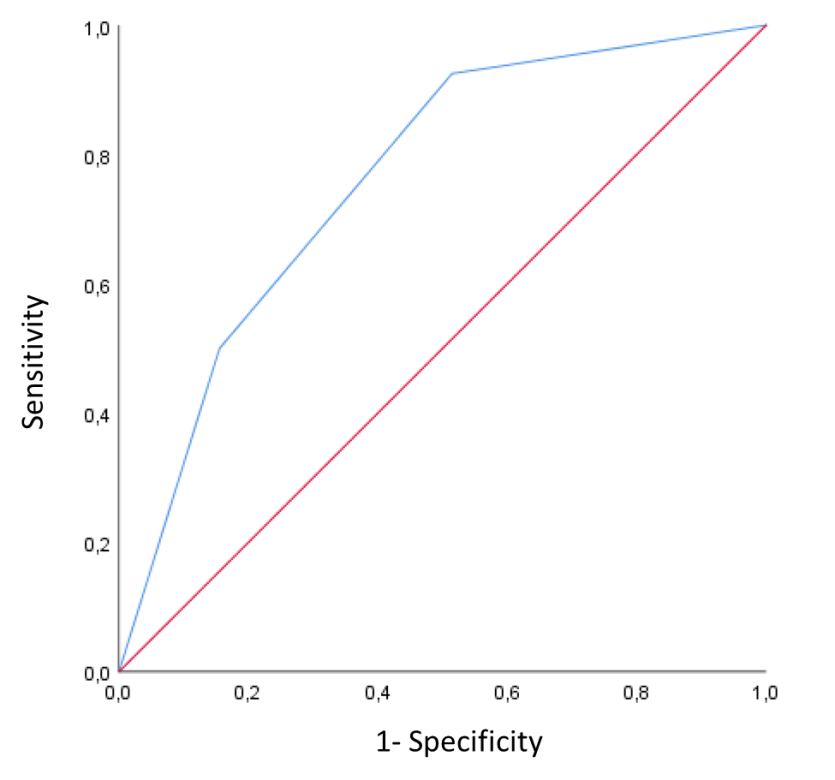


### Figure E2. AUC of independent predictor – Mean pulse pressure 24 hours after eCPR

AUC = 0.76.
